# Clarifying the Configuration of Pandamine by an Extensive Spectroscopic Reinvestigation of the Authentic 1964 Sample

**DOI:** 10.3390/metabo13040470

**Published:** 2023-03-24

**Authors:** Pascal Retailleau, Evodie Numbi Wa Ilunga, Véronique Fontaine, Jean-François Gallard, Pierre Le Pogam

**Affiliations:** 1 Institut de Chimie des Substances Naturelles, CNRS, ICSN UPR 2301, Université Paris-Saclay, 91198 Gif-sur-Yvette, France; 2 Microbiology, Bioorganic and Macromolecular Chemistry Unit, Université Libre de Bruxelles (ULB), Faculty of Pharmacy, Campus Plaine, Boulevard du Triomphe, 1050 Bruxelles, Belgium; 3 Équipe “Chimie des Substances Naturelles” BioCIS, CNRS, Université Paris-Saclay, 17 Avenue des Sciences, 91400 Orsay, France

**Keywords:** structure reassessment, peptide alkaloid, ansapeptide, pandamine, pandaceae

## Abstract

Since its partial configurational assignment in 1964, pandamine has not been isolated or obtained by total synthesis. For decades, different works representing the structure of pandamine for illustrative purposes have lent different configurations to this molecule, causing tenacious confusion about the structure of this ansapeptide. A comprehensive spectroscopic analysis of the authentic pandamine sample led to the complete and unambiguous assignment of its configuration, 59 years after its isolation. In addition to ascertaining and completing the initial structural deductions by a state-of-the-art set of analytical techniques, the purpose of this study is also to clarify the literature in a context in which various erroneous structures have been attributed to pandamine for half a century. While fully in agreement with Goutarel’s conclusions, the specific example of pandamine should serve as a cautionary tale to any chemist interested in natural products, encouraging access to initial structural assignments rather than relying solely on subsequent, possibly erroneous, structure depictions of a natural product.

## 1. Introduction

Pandamine (**1**), obtained from the crude alkaloid fraction of *Panda oleosa* Pierre root bark (Pandaceae) [1,2], represents the first cyclopeptidic alkaloid ever elucidated [3,4]. Since then, cyclopeptide alkaloids have been proven to be widespread plant metabolites but pandamine still holds a specific importance in this vast phytochemical class, marking the beginning of a considerable interest in these natural products, which now encompass more than 500 entries [5,6]. It is noteworthy that Goutarel et al. first proposed the term peptide alkaloids when they reported on the isolation of pandamine (**1**) in 1964 [1,5]. As such, publications reporting on the total synthesis of cyclopeptide alkaloids often refer to pandamine (**1**) [7,8,9,10], even though this illustrious lead has not yet succumbed to total synthesis.

**Figure 1:**
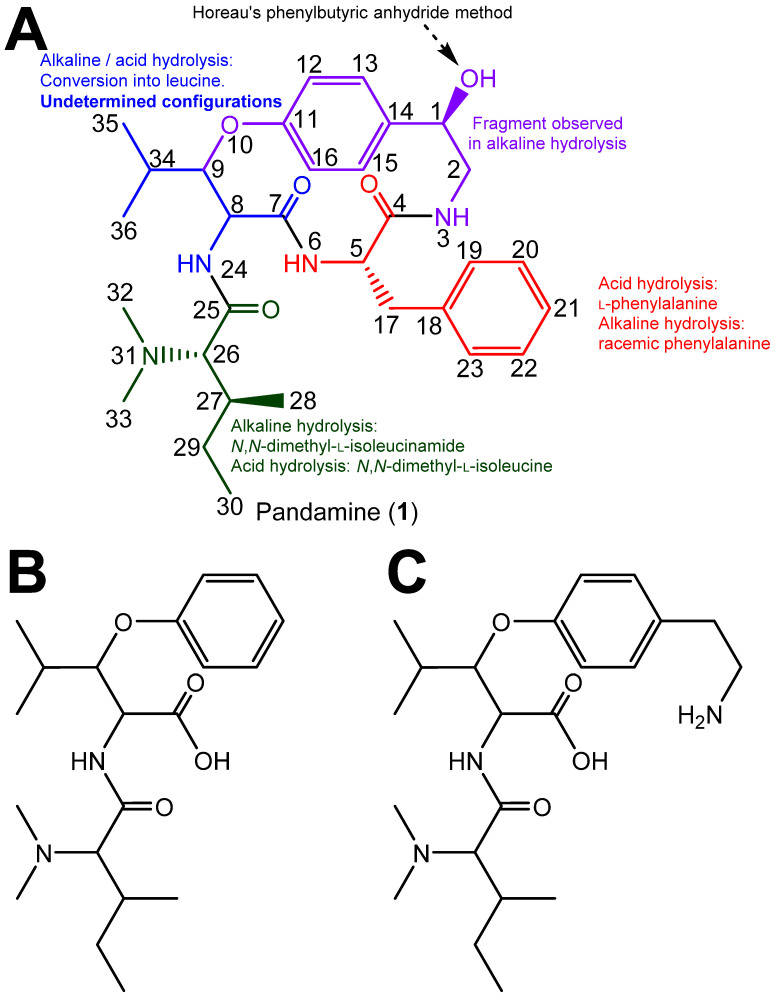
(**A**). Chemical structure of pandamine as determined by Goutarel, and supportive analytical evidence for highlighted fragments. (**B**,**C**). Key acid hydrolysis fragments observed from pandamine (**B**) and 1-deoxypandamine (**C**).

The structure determination of pandamine (**1**), undertaken by Goutarel et al. in the mid-1960s, is a masterful example of structural chemical sleuthing, chiefly involving chemical methods [1,2]. By then, the use of ^13^C NMR spectroscopy and the 2D NMR sequences would not become widely available until two decades later. In short, the alkaline hydrolysis of pandamine yielded a fragment identified as *N*,*N*-dimethyl-l-isoleucinamide (identified as such by comparison against a synthetic specimen), a racemic phenylalanine, a racemic leucine and a 2-paradihydroxyphenylethylamine. Combined together, these products accounted for all of the carbon atoms in the structure (Figure 1). Acid hydrolysis of pandamine provided some information on the linkage of these fragments. In particular, the identification of 1B as a cleavage product upon acid hydrolysis determined that the *N*,*N*-dimethylisoleucine and the β-hydroxyleucine were connected, and established that the oxygen of the β-hydroxyleucine residue was contained in the macrocycle. Fragment 1C, obtained after acid hydrolysis of the closely related 1-deoxypandamine, left only one possibility to incorporate the co-identified l-phenylalanine residue, convincingly reconstituting the final gross structure of pandamine, as indicated in Figure 1, in line with the determined molecular formula [2]. Optical rotations of the phenylalanine and *N*,*N*-dimethylleucine residues obtained by acid hydrolysis allowed the assignment of the l-stereochemistry of these amino acids. The *R* configuration of the C-1 stereocenter had been assigned on the basis of the so-called Horeau’s phenylbutyric anhydride method [2]. This insightful approach is based on the reaction of optically active secondary alcohols of unknown configuration with an excess of racemic 2-phenylbutyric acid, which proceeds at different rates to the diastereoisomeric esters. Horeau inferred that the absolute configuration of the alcohol could be derived from the enantiomeric ratio of the remaining 2-phenylbutyric acid [11]. Although interesting, this strategy presents inherent limitations that may lead to erroneous conclusions, especially in the case of small optical rotation values, which require the absence of any chiral impurities [12]. Conversely, the configurations of the two asymmetric carbons of the β-hydroxyleucine could not be mitigated, as the free amino acid could not be recovered under either acidic or alkaline hydrolysis conditions [2,3]. The release of leucine from this amino acid is supposed to proceed via hydrolysis to α-ketoisocaproic acid and further transamination, as reported for other cyclopeptide alkaloids [13] (Figure 1). It appears that the structure elucidation of pandamine has not progressed since Goutarel’s seminal contributions, as pandamine does not appear to have been isolated or synthesised elsewhere since. The ^13^C NMR data of pandamine were provided in a 1979 study that collated such spectroscopic information for an array of structurally-diverse cyclopeptide alkaloids [14]. This did not lead to the elucidation of any further structural features.

**Figure 2:**
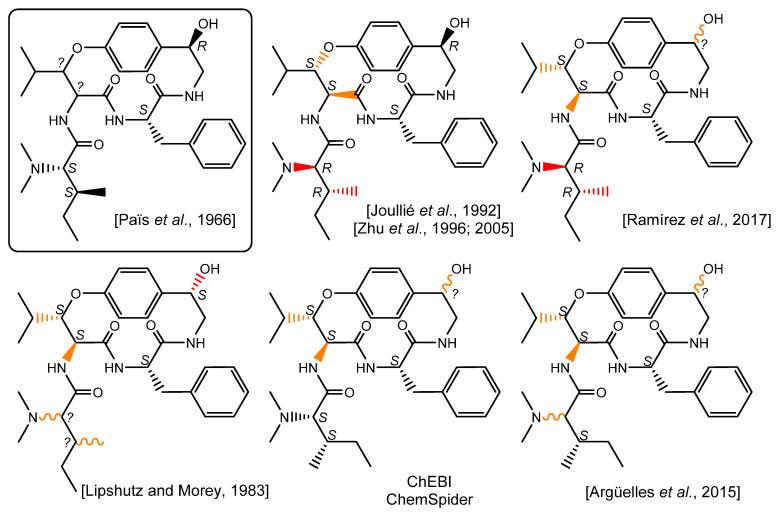
Chemical structure of pandamine as seminally elucidated in 1966 and a selection of later chemical depictions. Bonds appearing in red are inconsistent with some of the deductions made in the original structure elucidation report. Bonds appearing in orange do not find spectroscopic support OR do not take into account previously determined stereochemical assignments for wavy bonds. [2,7,8,9,15,16].

Surprisingly, further literature reports disclosed assigned configurations for the β-hydroxyleucine residue, which is often depicted as an l-erythro-β-OH-hydroxyleucine residue [4,8]. The analytical pieces of evidence supporting this claim are dubious as the references quoted either explicitly indicate that the configurations of these stereocenters remain unassigned [2] or are not discussed [14]. Although invariably referring to the same publication, most of the subsequent works disclosing the structure of pandamine retain a similar l-erythro-β-OH-hydroxyleucine. Besides, it is worth underlining that pandamine is represented with different chemical structures throughout the literature, often contradicting Goutarel’s seminal conclusions. For example, publications by the Joullié [8] and Zhu [9,10] groups disclose a surprising structure for pandamine incorporating a *N*,*N*-dimethyl-d-alloisoleucine moiety. A rather similar structure appeared in 2017, in which pandamine incorporates this latter erroneous amino acid, but does not propose a configuration for the C-1 secondary alcohol [15]. Conversely, a 1983 contribution by Lipshutz and Morey depicts an alternative structure for pandamine, revealing a stereochemically undefined *N*,*N*-dimethylisoleucine residue and a secondary alcohol with a configuration opposite to that initially determined in 1966 [7]. Further graphical depictions of this compound fail to take into account some duly assigned stereocenters, while disclosing an l-erythro-β-OH-hydroxyleucine residue with seemingly no spectroscopic support [16]. While these different chemical structures for pandamine are probably misrepresentations rather than genuine alternative candidates, the impact of these mistakes should not be underestimated, especially considering that the original articles, written in French, are very hard to access. In this context, these chemical structures are likely to be the first, if not the only, stereochemically-informed depictions of pandamine that interested readers will reach, especially when the Dictionary of Natural Products erroneously indicates that the pandamine structure is a « gross structure with no stereochemistry » [17], and the CAS entry for pandamine retains the structure depicted in the Joullié and Zhu publications, even though they partly contradict Goutarel’s deductions (Figure 2).

## 2. Materials and Methods

### 2.1. General Experimental Procedures

HRESIMS measurements used an Agilent 6546 Accurate-Mass Q-TOF, hyphenated with a 1290 Agilent Infinity II LC system (Agilent, Palo Alto, CA, USA). The chromatographic system was fitted with a Zorbax RRHD Eclipse Plus C_18_ column (2.1 × 50 mm, 1.8 μm). ^1^H and ^13^C NMR data were recorded on an AM-500 (500 MHz) Bruker 700 MHz NMR spectrometer (Bruker Daltonics, Bremen, Germany) using TFA-*d* or DMSO-*d*_6_ as solvent (Euriso-Top, Saint-Aubin, France).

Pandamine (**1**). White amorphous solid; mp = 256 °C; [α]_D_^25.0^ − 77 (*c* 1, CH_3_OH); IR *ν*_max_ 3300, 3050, 1650, 1600, 1540, 1500, 1250 cm^−1^; *λ*_max_ (log *ε*) 230 (3.59), 260 (1.80) and 285 (1.80); ^1^H and ^13^C NMR data, see Table 1; HR-ESI-MS *m/z* 553.3394 [M + H]^+^ (calculated for C_31_H_45_N_4_O_5_, 553.3384). MS/MS spectrum was deposited in the GNPS spectral library under the identifier CCMSLIB00010128917.

### 2.2. X-ray Crystallographic Analysis of Pandamine

High resolution crystallographic data for **1** were collected using a Rigaku XtaLabPro single-crystal diffractometer, equipped with a microfocus Mo K*α* radiation and a HPAD PILATUS3 R 200K detector. *CrysAlisPro 1.171.42.75a* [18] was employed for the processing of the redundant data recorded at low temperature (123K, under a stream of liquid nitrogen) from multiple scans, with a combination of empirical absorption correction using spherical harmonics, implemented in the SCALE3 ABSPACK scaling algorithm and numerical absorption correction based on Gaussian integration over a multifaceted crystal model. **1** crystallized in the trigonal Sohncke space group, P3_2_21, and its structure was readily solved by intrinsic phasing methods (*SHELXT*) [19], then refined by full-matrix least-squares methods on *F*^2^ using *SHELX-L* [20] with one copy in the asymmetric unit. The non-hydrogen atoms were refined anisotropically, and the hydrogen atoms, all identified in difference maps, were geometrically positioned for those bound to carbon atoms and refined using a riding model with U_iso_ set to xU_eq_ of the parent atom (x = 1.2, or 1.5 for methyl carbons). The hydrogens carried by the amide nitrogen atoms, N3 and N6, as well as the one carried by N31, had their positions freely refined with U_iso_ = 1.2U_eq_ (N). The same was also true for the hydrogens of a water molecule, which is duplicated in the unit cell to bridge two alkaloid molecules in a head-to-tail manner. This water, H-bonded to the nitrogen atom N31 of the protonated tertiary amine, is linked to the 2-fold symmetry-related adjacent pandamine molecule at the y, x, 1-z position, both directly via the carbonyl oxygen atom O7 and via a chlorine anion, h-bonded to the hydrogens carried by the amide N3 atom and the hydroxy O1 atom, respectively (Figure S15). The chlorine anion, which ensures the electroneutrality of the crystal, is at a distance of 3.907(2) Å from the protonated N3 atom. Pandamine molecules are arranged around a three-fold screw axis passing through the origin of the unit cell along the *c* axis, which, via its disordered phenyl group, delineates interstitial infinite channels in which the disordered crystallizing solvent (MeOH/CHCl_3_ (1/1, *v*/*v*)) could not be interpreted in the atomic pattern (Figure S2). It was therefore treated with the SQUEEZE routine as implemented in the *PLATON* program [21], and the procedure corrected for *ca* 35 electrons within solvent-accessible voids of *ca* 664 Å^3^. With respect to these interstitial channels, the disordered phenyl group could display three different orientations with respect to those of a two-fold rotation axis-related molecule, whose respective occupancies converged to values of 0.61/0.27/0.12 and whose atoms were restrained to have similar geometries and similar *U*^ij^ components of ADPs. Pandamine belongs to the largest subclass of cyclopeptide alkaloids, namely *p*-phencyclopeptines, which is based on a 14-membered ring containing a *β*-hydroxyamino acid group linked through an aryl ether bond to a *p*-hydroxystyrylamine moiety, as illustrated by frangulanine [22]. In contrast to this natural product, the C1-C2 bond is saturated and C1 is substituted by a hydroxy group, and the isobutane group at C5 is replaced by a phenyl group. Only one structure of synthetic *p*-phencyclopeptine is available to date at the CSD [23] (RefCode BUCKOZ), although the absolute structure of tri-*N*-methylfrangulanine methiodide was published in 1976 [24,25]. The absolute configurations of the six chiral centers in pandamine (Figure S15C) were unambiguously confirmed as 1*R*, 5*S*, 8*S*, 9*S,* 26*S*, and 27*S* from the anomalous scattering by the chlorine ions using established methods [26,27,28]. Consistent with previously related published X-ray structures, the *p*-phencyclopeptine nucleus of pandamine adopts the *trans*, *trans* configuration in the crystal, which is related to the geometry of the two amides, with the carbonyls pointing towards opposite sides of the macrocycle (Figure S15D).

Single-crystal X-ray diffraction data, data collection and structure refinement details are summarized in Table S1. Crystallographic data for structure **1** have been deposited in the Cambridge Crystallographic Data Centre database (deposition number CCDC 2235441). Copies of the data can be obtained free of charge from the CCDC at www.ccdc.cam.ac.uk (accessed on 15 February 2023).

### 2.3. Antimicrobial Activity Assay by Microdilutions

Antimicrobial activity was assessed against *Staphylococcus aureus* (ATCC 6538), *Staphylococcus epidermidis* (ATCC 14990), *Pseudomonas aeruginosa* (ATCC 15442) and *Candida albicans* (ATCC 64550) as previously described [29]. Assays were performed in 96-well plates using a broth microdilution method to determine the minimum inhibitory concentration (MIC). Two-fold serial dilutions (100 µL in 200 µL final volume) of the molecules were first performed in triplicate in Mueller Hinton broth. Then, 100 μL bacteria (100-fold dilution of 0.5 Mc Farland inoculum) were added into each well. Negative (no bacteria) and positive (no additional compounds) controls were included in each plate. The MIC values were first interpreted after 24 h incubation at 37 °C (corresponding to the lowest concentration inhibiting bacteria or yeast growth after visual inspection). This visual interpretation was eventually verified by adding MTT reagent, 3-(4,5-dimethylthiazol-2-yl)-2,5-diphenyltetrazolium bromide, (0.5 mg/mL final concentration) to allow formazan crystal formation in viable cells during an additional 4 h incubation period. A final visual inspection was performed to record the results. This assay was performed in triplicate in three separate experiments. Positive control drugs were vancomycin hydrochloride (Sigma, purity ≥ 900 μg/mg) for *S. aureus* and *S. epidermidis*, ofloxacin (Sigma, purity ≥ 99%) for *P. aeruginosa* and caspofungin (Cancidas, MSD) for *C. albicans*.

### 2.4. Cytotoxicity Assessment

The cytotoxicity of pandamine (**1**) was investigated in the human epithelial cell line, SiHa, in a MTT assay. The human cervical cancer cell line HTB-35 (ATCC designation, HTB-35, SiHa) was purchased from the American Type Cell Culture collection. Briefly, cells (1.10^5^/mL) were seeded in a 96-well plate at 100 µL/well and incubated at 37 °C with 5% CO_2_ for 24 h. Two-fold serial dilutions of compounds (in same culture DMEM-10% bovine serum) were added (100 µL/well) to the adherent SiHa cells and further incubated for 2 days at 37 °C. MTT reagent (0.5 mg/mL final concentration) was added to each well, and 4 h later, the wells were washed twice with PBS (phosphate buffer saline) and formazan crystals were dissolved in 100 µL/well DMSO. Absorbance was measured at 570 nm and 630 nm using a microplate reader (Bio-Rad 680) to assess cell viability. Results were interpreted by comparison with the optical density (OD) values from the control cells, without compounds (set to 100% viability). The positive control drug was orlistat (Sigma, purity ≥ 98%), which gave a 50% inhibitory concentration at 70 µg/mL, as previously described in various cell lines [30].

## 3. Results and Discussion

**Figure 3:**
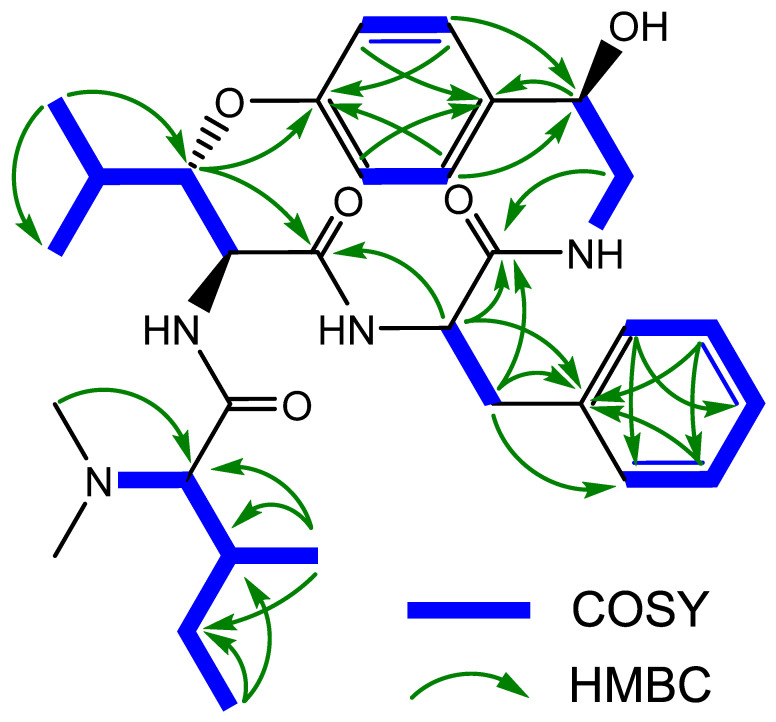
Key 2D NMR correlations of pandamine (**1**).

The persistent contradictions in the literature concerning the structure of pandamine and the doubts about the spectroscopic data underlying the assignment of certain asymmetric centers in the structure call for a new spectroscopic study of this compound. In addition, such a reinvestigation would provide a convenient opportunity to acquire an up-to-date set of spectroscopic information, thus securing the formerly assigned configurations of pandamine (**1**). As part of our continuing interest in re-evaluating the structure of historical compounds from our laboratories [31,32,33], we could locate the original sample of pandamine (**1**) to embark on a new, extensive spectroscopic analysis using current state-of-the-art spectroscopic techniques. The conclusions of this spectroscopic reinvestigation are outlined below. The UHPLC-DAD-HRMS^2^ analysis of the 1964 pandamine sample revealed a single signal in both UV and mass spectrometric detection. The unique peak appearing in the mass spectrometric chromatogram revealed a signal at *m/z* 553.3394, consistent with the awaited elemental composition of C_31_H_44_N_4_O_5_. We acquired the 1D and 2D NMR data, which confirmed its planar structure and permitted the complete assignment of all ^1^H and ^13^C NMR signals (Table 1) (Figure 3).

Particular attention has been paid to the signals associated with the stereochemically-undefined β-hydroxyleucine residue. The magnitude of the vicinal coupling constants related to the β-methine of the oxyleucine residue H-9 (*δ*_H_ 4.69, dd, *J* = 9.0, 1.8 Hz) were strongly reminiscent of those observed in 14-membered ansapeptides featuring an erythro-β-hydroxyleucine, hinting that pandamine should include a similar relative configuration for this residue [34] (a *threo* configuration would be expected to result in a *J*_8,9_ coupling of about 2 Hz) [35]. Likewise, the cursory analysis of the ^13^C NMR chemical shifts related to the α and β-methines for the l-erythro and d-erythro β-OH amino acids incorporated in 14-membered *para* ansapeptides disclosed significant differences between them. The presently determined chemical shifts of C-8 and C-9 were indicative of an l-erythro-β-hydroxyleucine residue [35]. These spectroscopic landmarks provided strong spectroscopic support for the commonly depicted configuration of this residue (Figure 2), despite the apparent lack of direct evidence. The cursory analysis of the NMR signals related to the hydroxystyrylamine side chain were strongly reminiscent of those of sanjoinine G1, where the 1*R* absolute configuration had been assigned on the basis of the exciton-coupled circular dichroism [36]. The magnitude of the vicinal coupling constants related to the diastereotopic methylenic protons H-2 and the ROE crosspeak between H-1 and the more unshielded proton of this methylenic pair supported an identical 1*R* configuration, as seminally determined by Horeau’s method [2].

**Figure 4:**
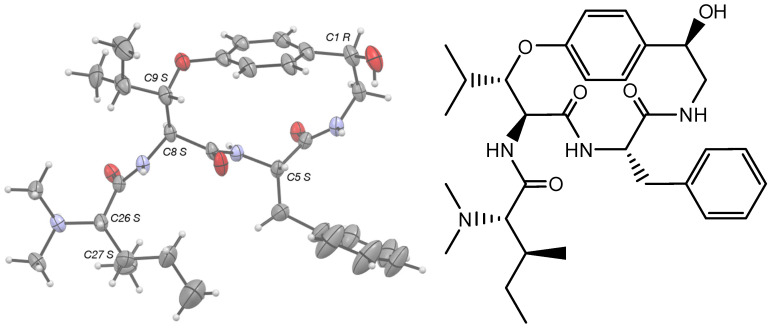
Structure and X-ray ORTEP representation of pandamine (**1**).

Gratifyingly, pandamine (**1**) could be crystallized from MeOH/CHCl_3_ (1/1, *v/v*). The single-crystal X-ray diffraction experiment validated the stereochemical assignments proposed in the Goutarel publication: l-phenylalanine, an *N*,*N*-dimethyl-l-isoleucine and the *R* configuration of the secondary alcohol. Besides, the ORTEP diagram validated the occurrence of a β-erythro-hydroxy-l-leucine, which has been widely reported but has so far been difficult to assign to a spectroscopic substratum (Figure 4).

The in vitro antibacterial activities of pandamine (**1**) were evaluated against *Staphylococcus aureus*, *S. epidermidis* and *Pseudomonas aeruginosa*. Pandamine (**1**) was inactive against all bacteria tested in the concentration range evaluated (0.75–50 µg/mL). Pandamine (**1**) also revealed no antifungal activity against *Candida albicans* neither. Likewise, pandamine (**1**) exerted no cytotoxicity towards SiHa cells in the range of 0.75–50 µg/mL.

## 4. Conclusions

Although this study is an analytical clarification rather than a true structural revision, it should be emphasised that the retained configuration differs from all stereochemically-informed pandamines found in the literature. The elevated number of misrepresentations of pandamine is alarming, but is probably related to the difficult access to the first French publications, which facilitated the dissemination of errors made in early representations of the pandamine structure.

## Figures and Tables

**Table 1:** ^1^H and ^13^C NMR data (500/125 MHz) for pandamine (**1**).

	Recorded in DMSO-*d*_6_		Recorded in TFA-*d*	
	*δ*_H_ (*J* in Hz)	*δ*_C_	*δ*_H_ (*J* in Hz)	*δ*_C_
1	4.89 (br s)	70.6	5.27 (d, 3.9)	75.7
1-OH	5.21 (br s)			
2α	3.96 (ddd, 13.7, 10.7, 4.4)	47.3	4.19 (dd, 15.1, 4.0)	49.4
2β	2.73 (d, 13.7)		3.07 (d, 15.1)	
3	7.27 (d, 10.7)			
4		169.0		173.2
5	4.27 (dt, 6.1, 8.3)	53.0	4.51 (dd, 8.7, 6.6)	57.8
6	7.47 (br s)			
7		168.5		173.0
8	4.51 (m)	54.9	4.84 (d, 9.2)	58.5
9	4.69 (dd, 9.0, 1.8)	79.1	4.78 (d, 9.2)	79.7
11		155.5		159.4
12	6.70 (dd, 8.3, 2.5)	118.5	6.70 (dd, 8.6, 2.4)	121.0
13	6.86 (dd, 8.3, 2.0)	126.6	6.97 (dd, 8.6, 1.8)	130.0
14		134.9		132.3
15	7.25 (dd, 8.8, 2.0)	126.5	7.18 (dd, 8.4, 2.1)	128.4
16	6.81 (dd, 8.8, 2.5)	113.4	6.82 (dd, 8.8, 2.5)	114.0
17	2.35 (dd, 13.8, 6.2) 2.63 (dd, 13.8, 8.3)	39.0	2.74 (dd, 13.6, 8.4) 2.60 (dd, 13.6, 6.4)	41.5
		
18		136.8		136.2
19	7.02 (m)	128.8	6.91 (dd, 6.6, 1.7)	130.9
20	7.16 (m)	127.8	7.11 (m)	130.8
21	7.11 (t, 7.3)	126.0	7.09 (ov)	129.7
22	7.16 (m)	127.8	7.11 (m)	130.8
23	7.02 (m)	128.8	6.91 (dd, 6.6, 1.7)	130.9
24				
25		n.d.		168.2
26	3.73 (br s)	70.2	3.97 (d, 4.4)	75.5
27	1.94 (m)	33.0	2.11 (m)	36.7
28	0.72 (d, 6.4)	13.4	0.84 (d, 6.9)	13.6
29	0.99 (ov.), 1.49 (m)	25.3	0.92 (m), 1.38 (m)	28.6
30	0.83 (t, 7.3)	10.9	0.92 (t, 7.2)	12.4
32	2.71 (s)	41.2	3.01 (s)	42.8
33	2.71 (s)	41.2	3.05 (s)	45.6
34	2.13 (m)	27.9	1.97 (hept, 6.6)	31.3
35	1.07 (d, 7.3)	20.1	1.05 (d, 6.7)	20.3
36	0.95 (d, 6.7)	14.4	0.96 (d, 6.7)	14.9

## Data Availability

The data presented in this study are available in supplementary material.

## Supplementary Materials

The following supporting information can be downloaded at: https://www.mdpi.com/article/10.3390/metabo13040470/s1, Figure S1: (+)-HRESIMS analysis of pandamine (**1**); Figure S2: ^1^H NMR (DMSO-*d*_6_, 500 MHz) spectrum of pandamine (**1**); Figure S3: ^13^C NMR (DMSO-*d_6_*, 125 MHz) spectrum of pandamine (**1**); Figure S4: COSY (DMSO-*d*_6_, 500 MHz) spectrum of pandamine (**1**); Figure S5: COSY-TOCSY (DMSO-*d*_6_, 500 MHz) spectrum of pandamine (**1**); Figure S6: Edited HSQC (DMSO-*d*_6_, 500/125 MHz) spectrum of pandamine (**1**); Figure S7: HSQC-TOCSY (DMSO-*d*_6_, 500/125 MHz) spectrum of pandamine (**1**); Figure S8: HMBC (DMSO-*d*_6_, 500/125 MHz) spectrum of pandamine (**1**); Figure S9: ROESY (DMSO-*d*_6_, 500 MHz) spectrum of pandamine (**1**); Figure S10: ^1^H NMR (TFA-*d*, 500 MHz) spectrum of pandamine (**1**); Figure S11: ^13^C NMR (TFA-*d*, 125 MHz) spectrum of pandamine (**1**); Figure S12: COSY (TFA-*d*, 500 MHz) spectrum of pandamine (**1**); Figure S13: (A) Edited HSQC (TFA-d, 500/125 MHz) spectrum of pandamine (**1**), (B) HMBC (TFA-d, 500/125 MHz) spectrum of pandamine (**1**).; Figure S14: ROESY (TFA-*d*, 500 MHz) spectrum of pandamine (**1**); Figure S15: X-ray crystallography related data, Table S1: Crystal data and structure refinement for pandamine (**1**).
