# Supplementary material for: Clarifying the Configuration of Pandamine by an Extensive Spectroscopic Reinvestigation of the Authentic 1964 Sample
Source: Metabolites. 2023 Mar 24;13(4):470. doi: 10.3390/metabo13040470 (PMC10147048; doi:10.3390/metabo13040470)
Supplement: Supplementary file 1 [file metabolites-13-00470-s001.zip › metabolites-2259071-supplementary.pdf]

metabolites

MDPI

Supplementary Materials

Clarifying the Configuration of Pandamine by an Extensive Spectroscopic Reinvestigation of the Authentic 1964 Sample

Pascal Retailleau [1], Evodie Numbi Wa Ilunga [2], Véronique Fontaine [2], Jean-François Gallard [1] and Pierre Le Pogam [3,*]

[1] Institut de Chimie des Substances Naturelles, CNRS, ICSN UPR 2301, Université Paris-Saclay, 91198 Gif-sur-Yvette, France; pascal.retailleau@cnrs.fr (P.R.); jean-francois.gallard@cnrs.fr (J.-F.G.)
[2] Microbiology, Bioorganic and Macromolecular Chemistry Unit, Université libre de Bruxelles (ULB), Faculty of Pharmacy, Campus Plaine, Boulevard du Triomphe, 1050 Bruxelles, Belgium; evodienumbi@gmail.com (E.N.W.I.); veronique.fontaine@ulb.be (V.F.)
[3] Équipe "Chimie des Substances Naturelles" BioCIS, CNRS, Université Paris-Saclay, 17 Avenue des Sciences, 91400 Orsay, France
* Correspondence: pierre.le-pogam-alluard@universite-paris-saclay.fr

TABLE OF CONTENTS

Figure S1. (+)-HRESIMS analysis of pandamine (**1**).
Figure S2. ^{1}H NMR (DMSO-d_6, 500 MHz) spectrum of pandamine (**1**).
Figure S3. ^{13}C NMR (DMSO-d_6, 125 MHz) spectrum of pandamine (**1**).
Figure S4. COSY (DMSO-d_6, 500 MHz) spectrum of pandamine (**1**).
Figure S5. COSY-TOCSY (DMSO-d_6, 500 MHz) spectrum of pandamine (**1**).
Figure S6. Edited HSQC (DMSO-d_6, 500/125 MHz) spectrum of pandamine (**1**).
Figure S7. HSQC-TOCSY (DMSO-d_6, 500/125 MHz) spectrum of pandamine (**1**).
Figure S8. HMBC (DMSO-d_6, 500/125 MHz) spectrum of pandamine (**1**).
Figure S9. ROESY (DMSO-d_6, 500 MHz) spectrum of pandamine (**1**).
Figure S10. ^{1}H NMR (TFA-*d*, 500 MHz) spectrum of pandamine (**1**).
Figure S11. ^{13}C NMR (TFA-*d*, 125 MHz) spectrum of pandamine (**1**).
Figure S12. COSY (TFA-*d*, 500 MHz) spectrum of pandamine (**1**).
Figure S13. (A) Edited HSQC (TFA-d, 500/125 MHz) spectrum of pandamine (**1**),<br>(B) HMBC (TFA-d, 500/125 MHz) spectrum of pandamine (**1**).
Figure S14. ROESY (TFA-*d*, 500 MHz) spectrum of pandamine (**1**).
Figure S15. X-ray crystallography related data

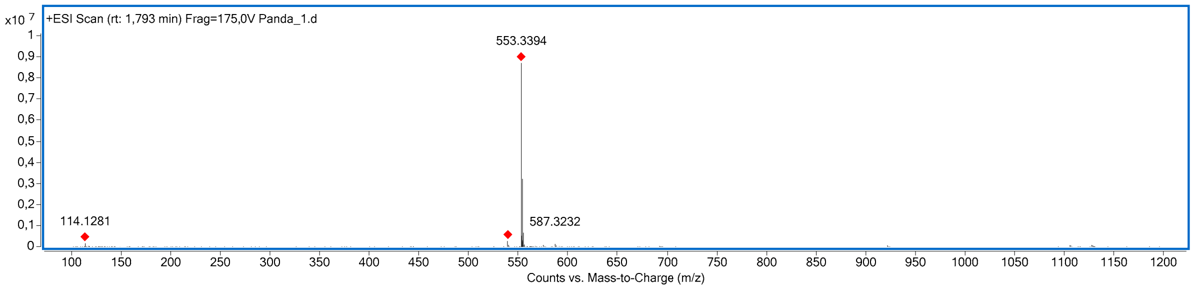

Figure S1. (+)-HRESIMS analysis of pandamine (**1**).

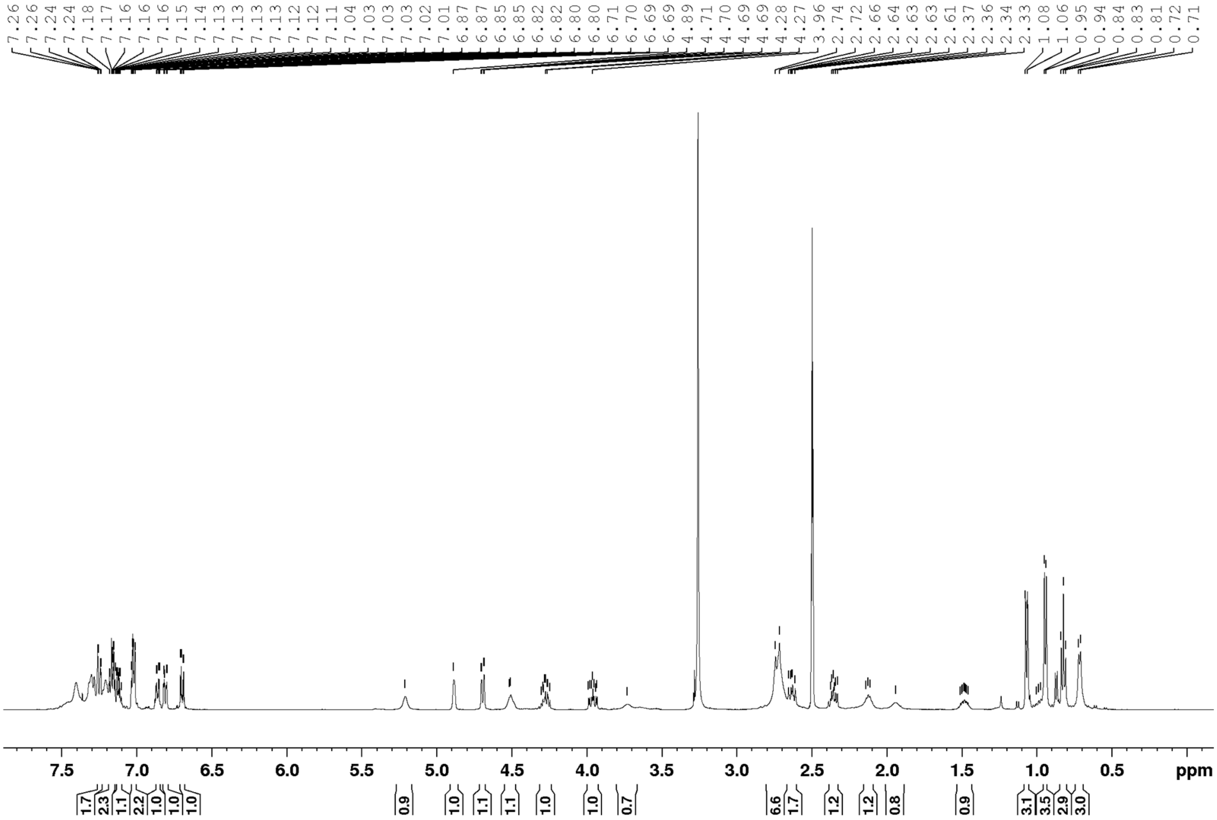

Figure S2. ^{1}H NMR (DMSO-d_6, 500 MHz) spectrum of pandamine (**1**).

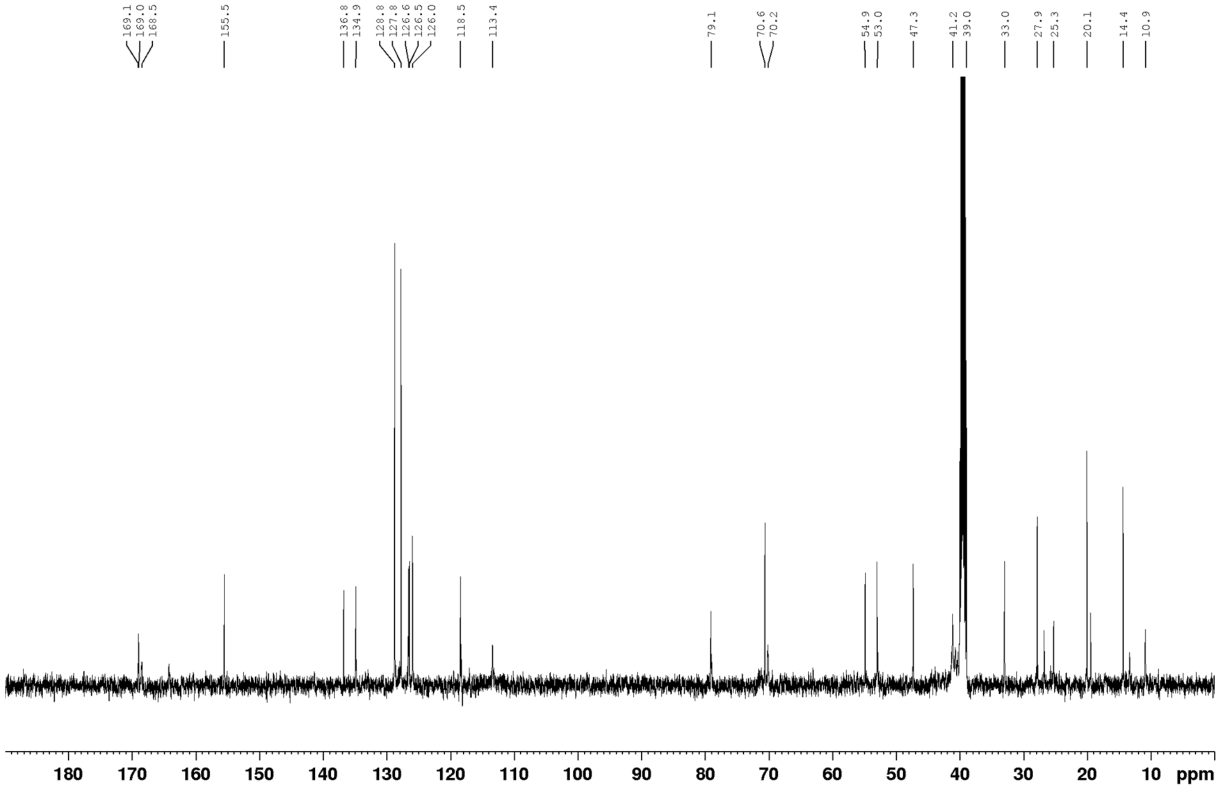

Figure S3. ^{13}C NMR (DMSO-d_6, 125 MHz) spectrum of pandamine (**1**).

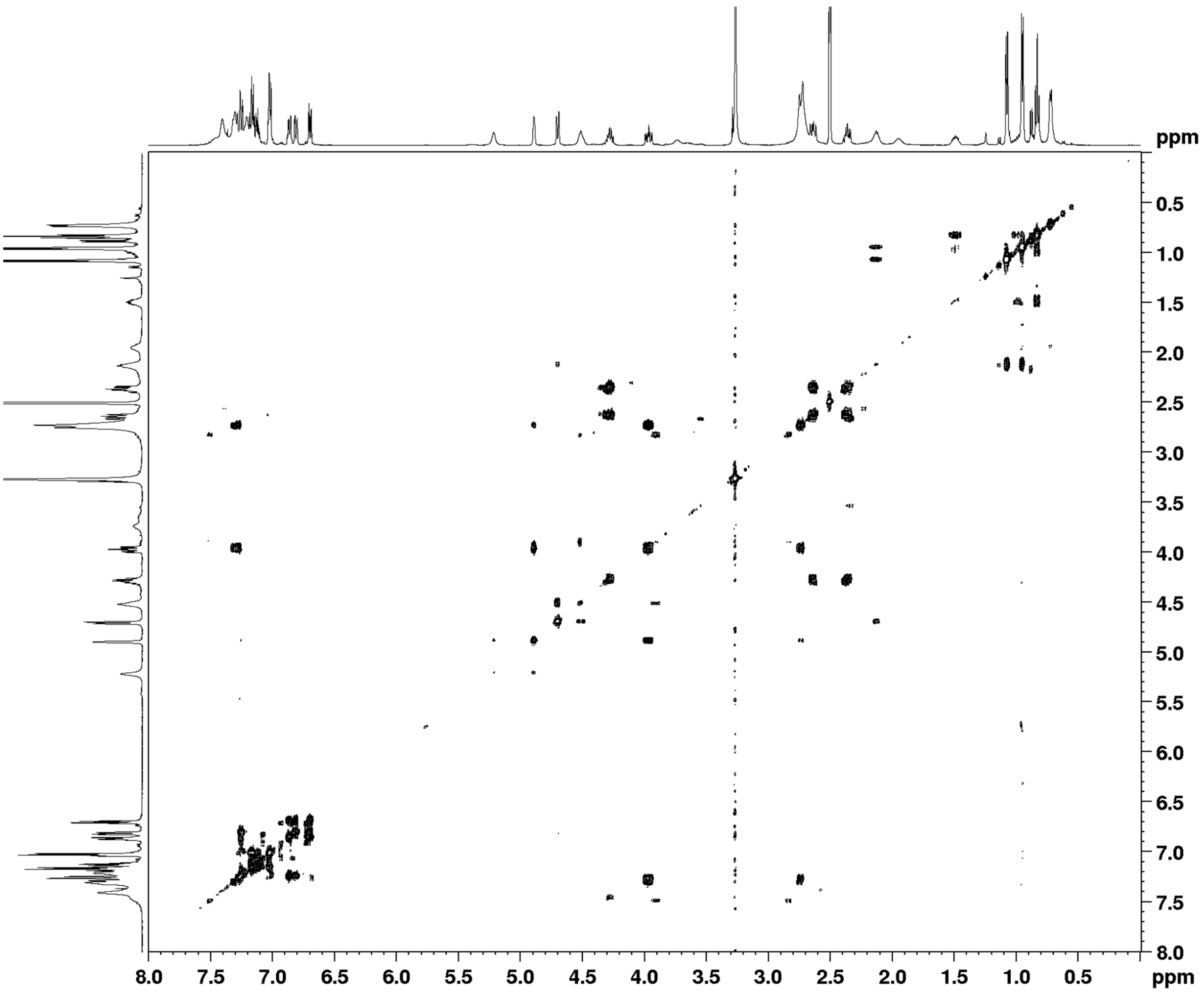

Figure S4. COSY (DMSO-d_6, 500 MHz) spectrum of pandamine (**1**).

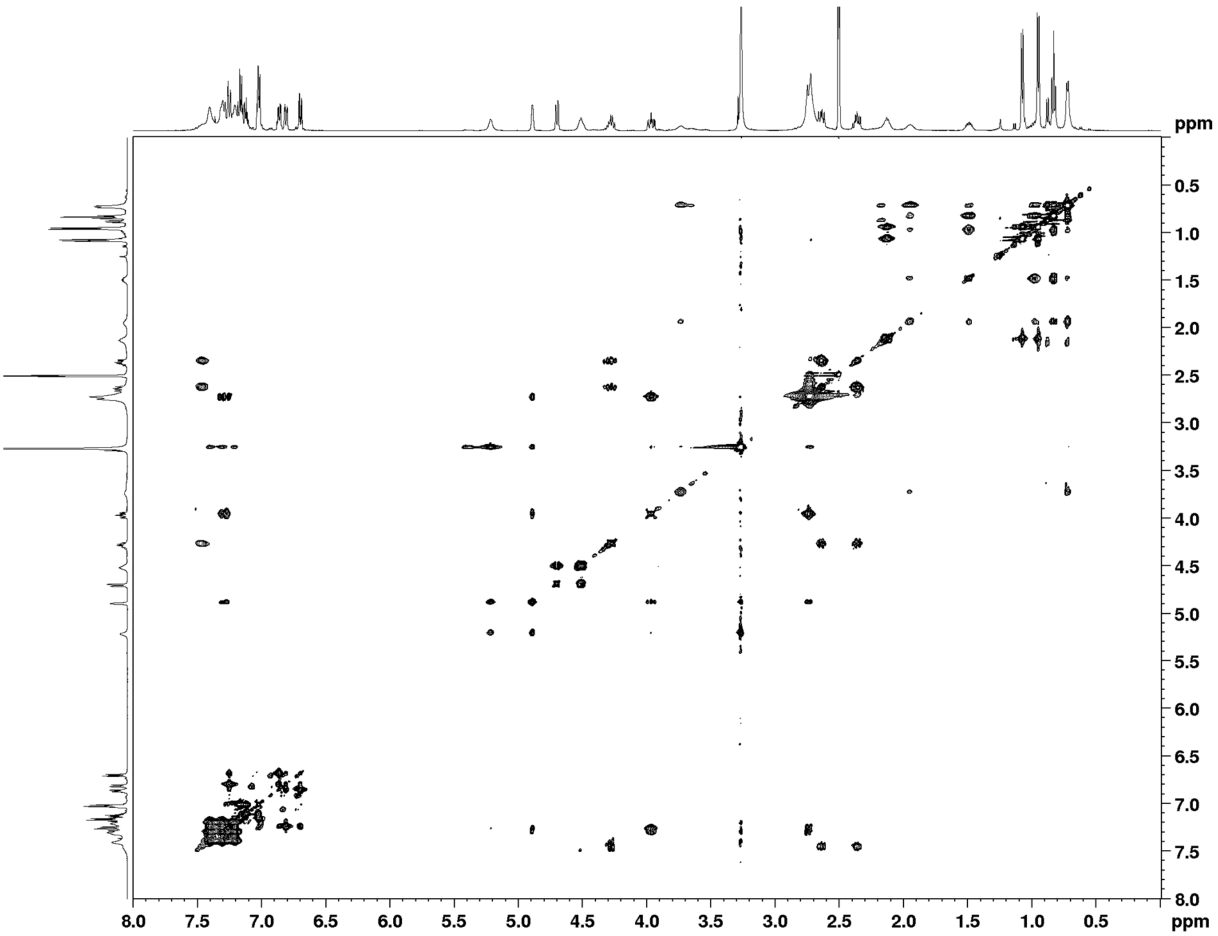

Figure S5. COSY-TOCSY (DMSO-d_6, 500 MHz) spectrum of pandamine (**1**).

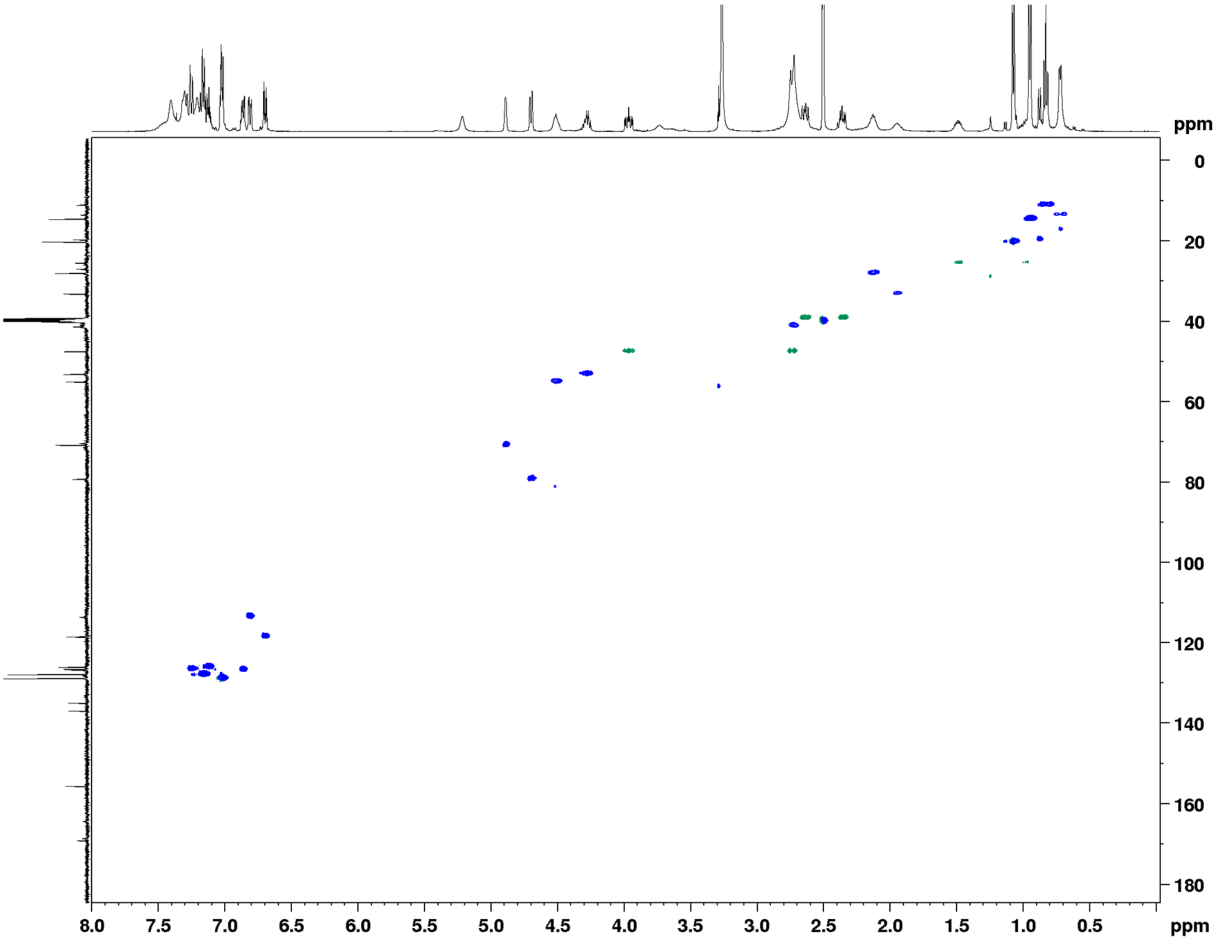

Figure S6. Edited HSQC (DMSO-*d*6, 500/125 MHz) spectrum of pandamine (**1**).

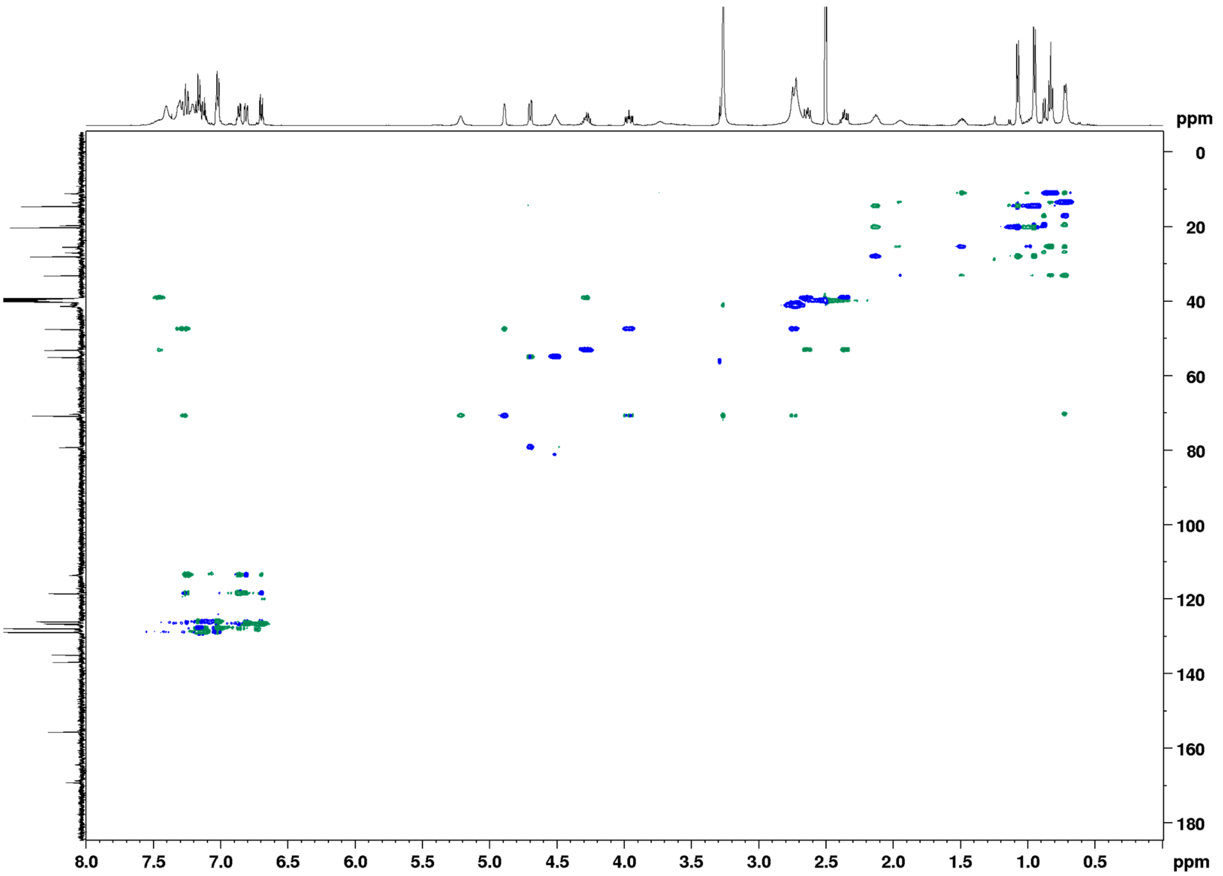

Figure S7. HSQC-TOCSY (DMSO-d_6, 500/125 MHz) spectrum of pandamine (**1**).

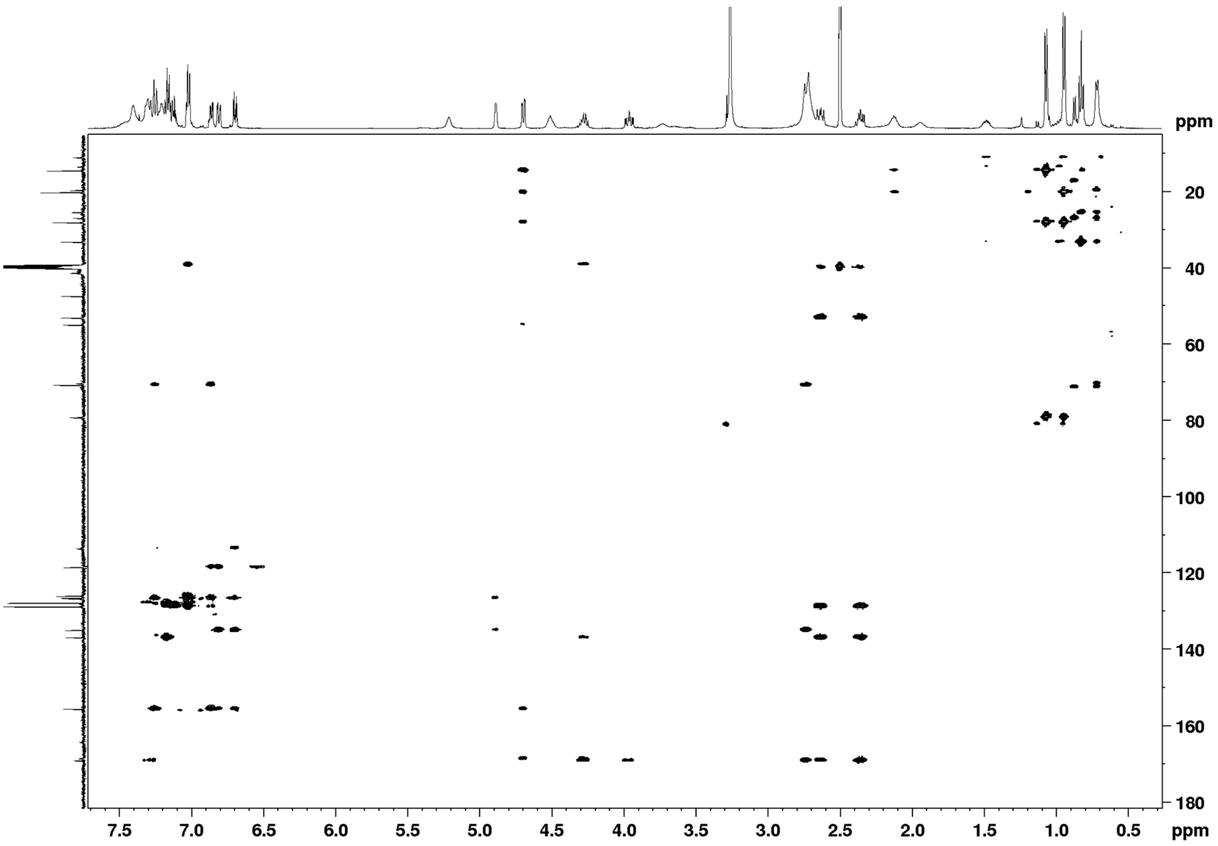

Figure S8. HMBC (DMSO-d_6, 500/125 MHz) spectrum of pandamine (**1**).

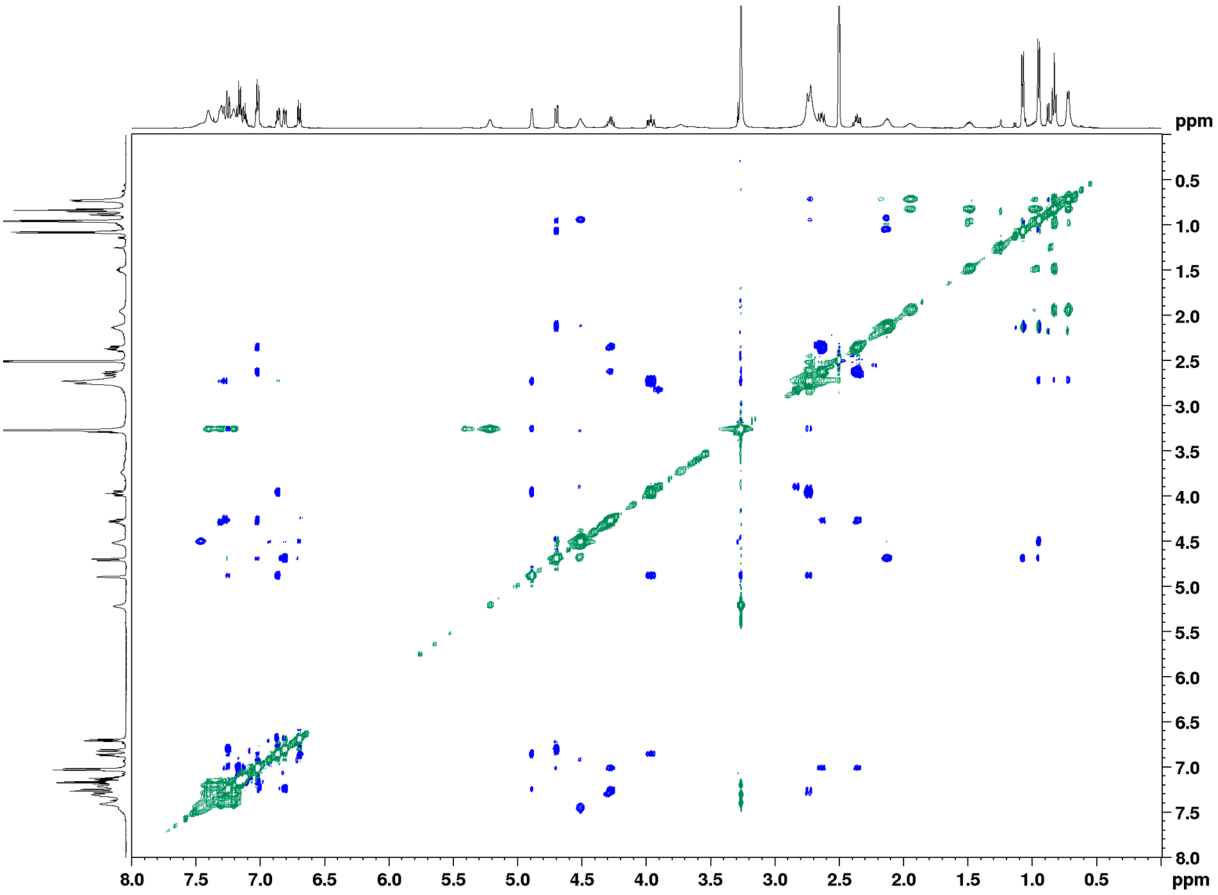

Figure S9. ROESY (DMSO-d_6, 500 MHz) spectrum of pandamine (**1**).

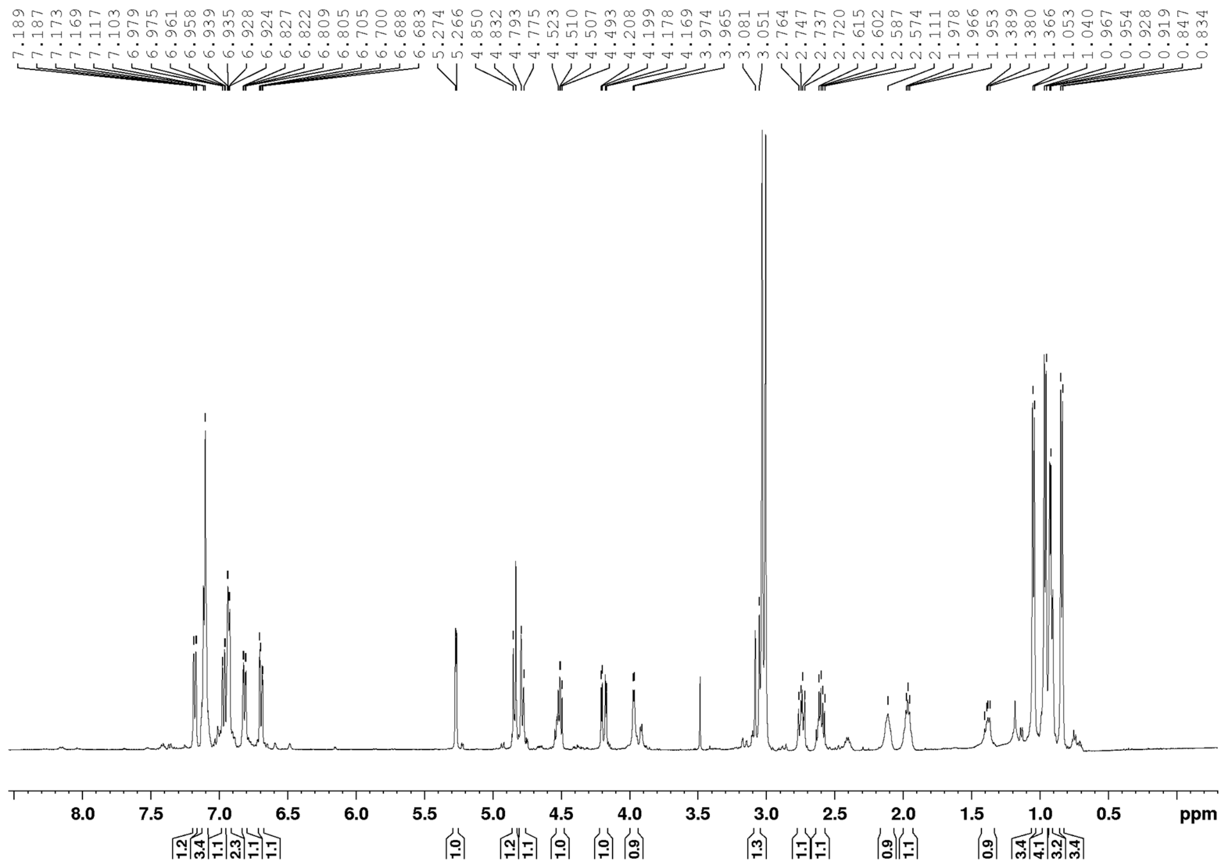

Figure S10. ^{1}H NMR (TFA-*d*, 500 MHz) spectrum of pandamine (**1**).

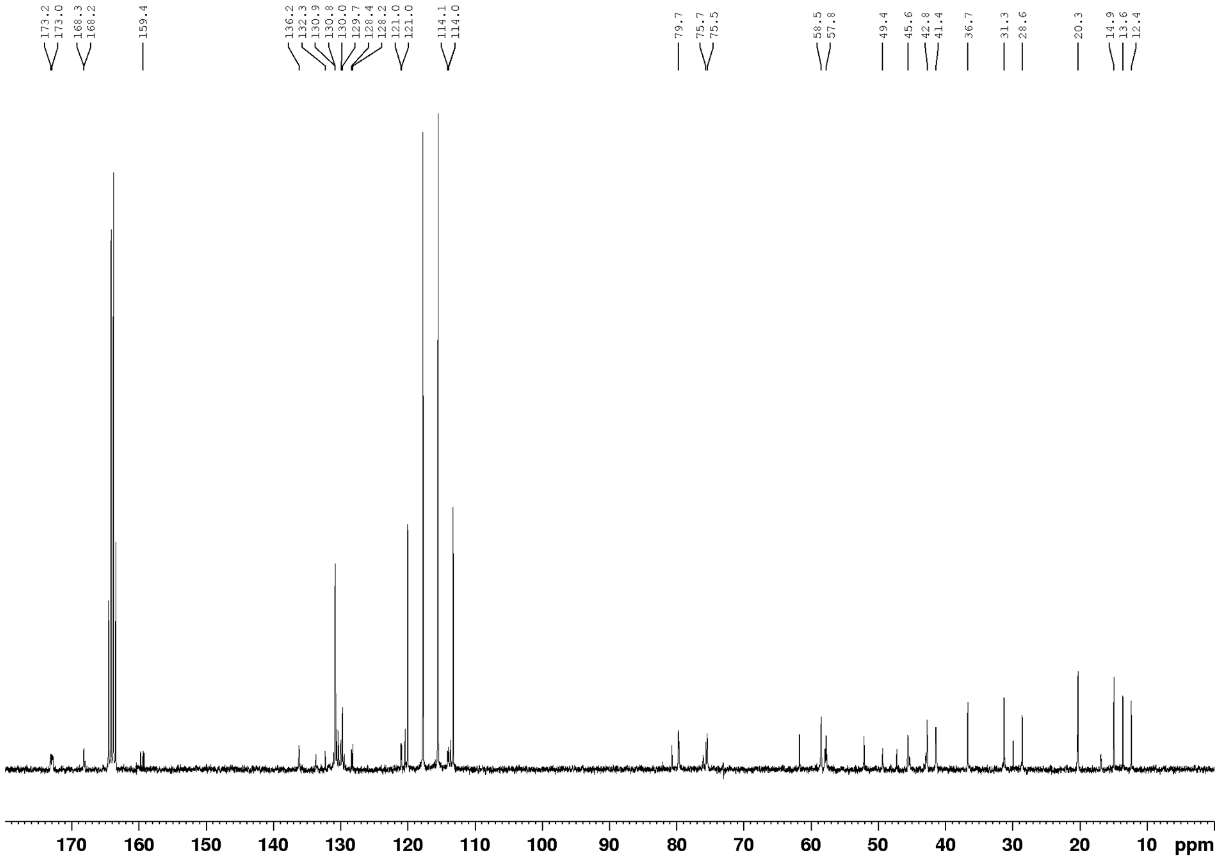

Figure S11. ^{13}C NMR (TFA-*d*, 125 MHz) spectrum of pandamine (**1**).

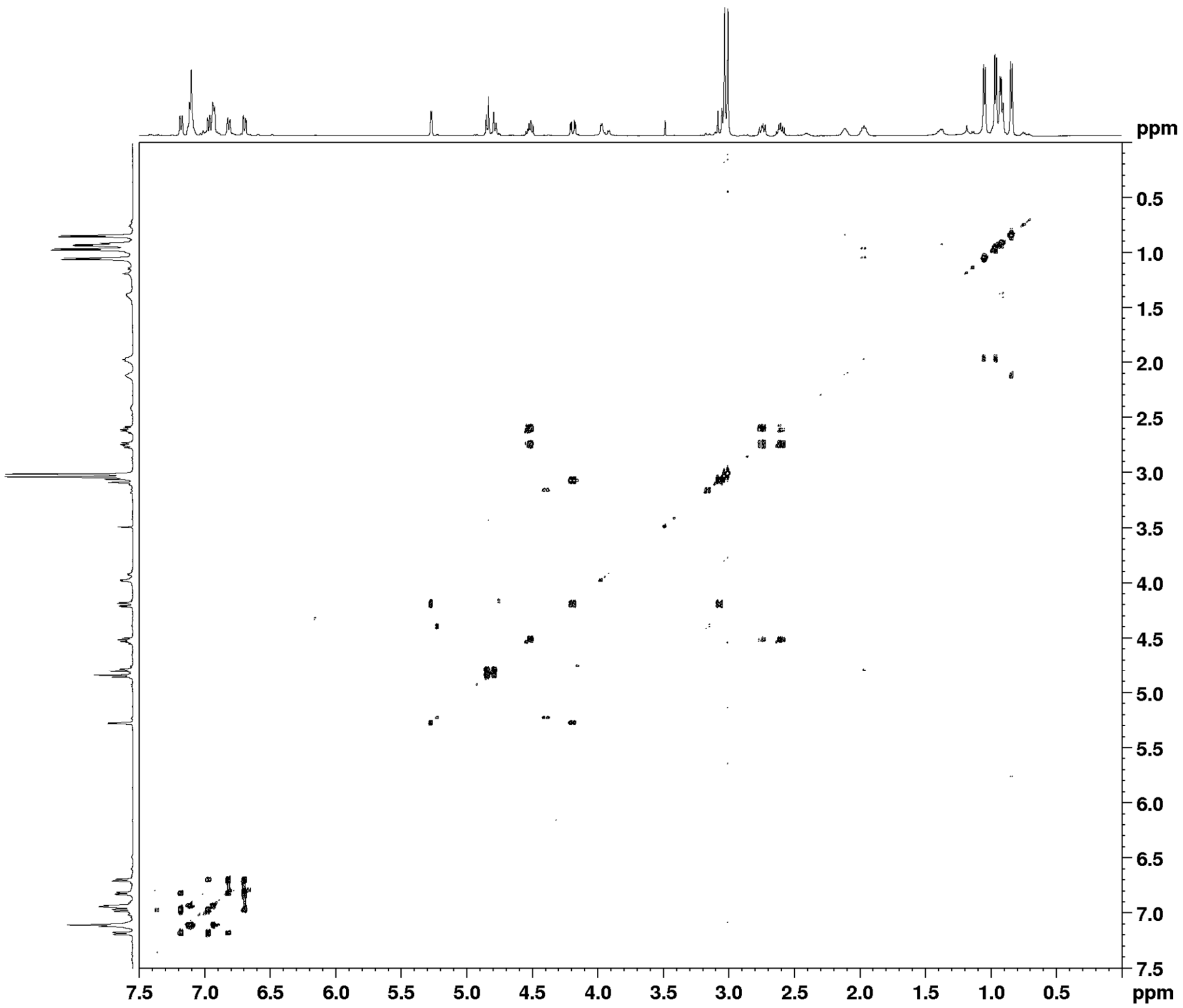

Figure S12. COSY NMR (TFA-*d*, 500 MHz) spectrum of pandamine (**1**).

(A)

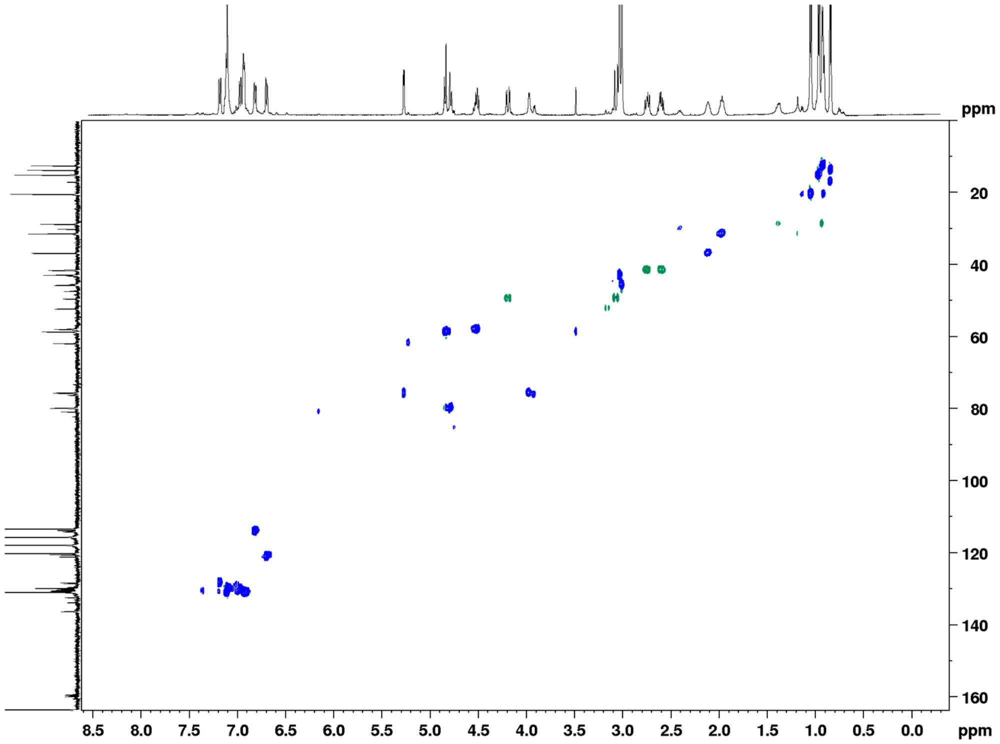

(B)

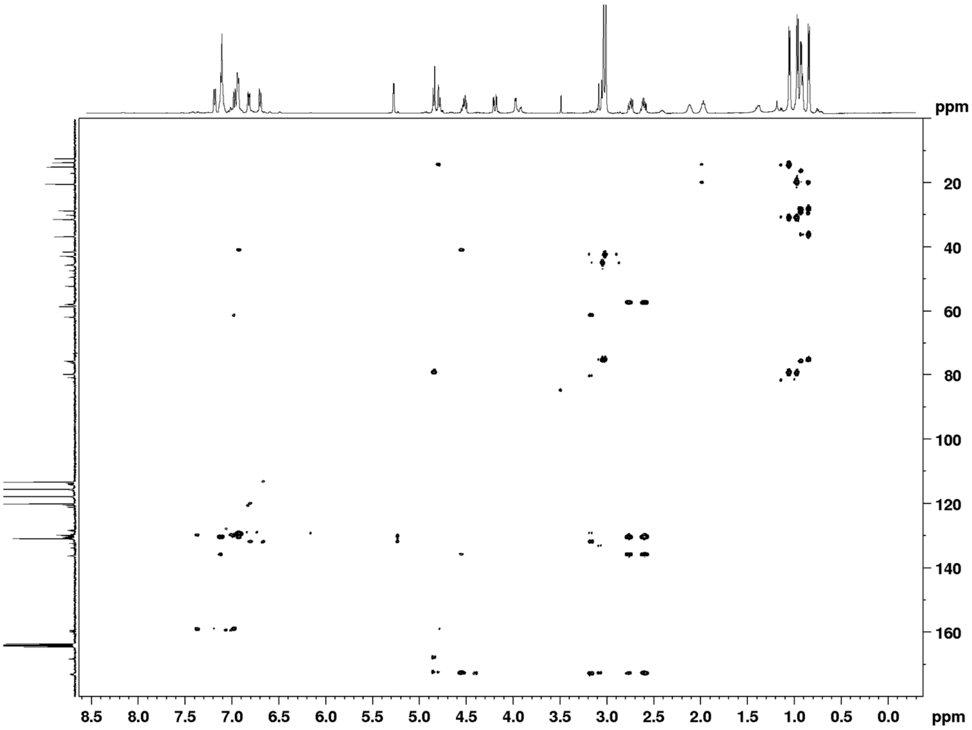

Figure S13. (**A**) Edited HSQC (TFA-*d*, 500/125 MHz) spectrum of pandamine (**1**), (**B**) HMBC (TFA-*d*, 500/125 MHz) spectrum of pandamine (**1**).

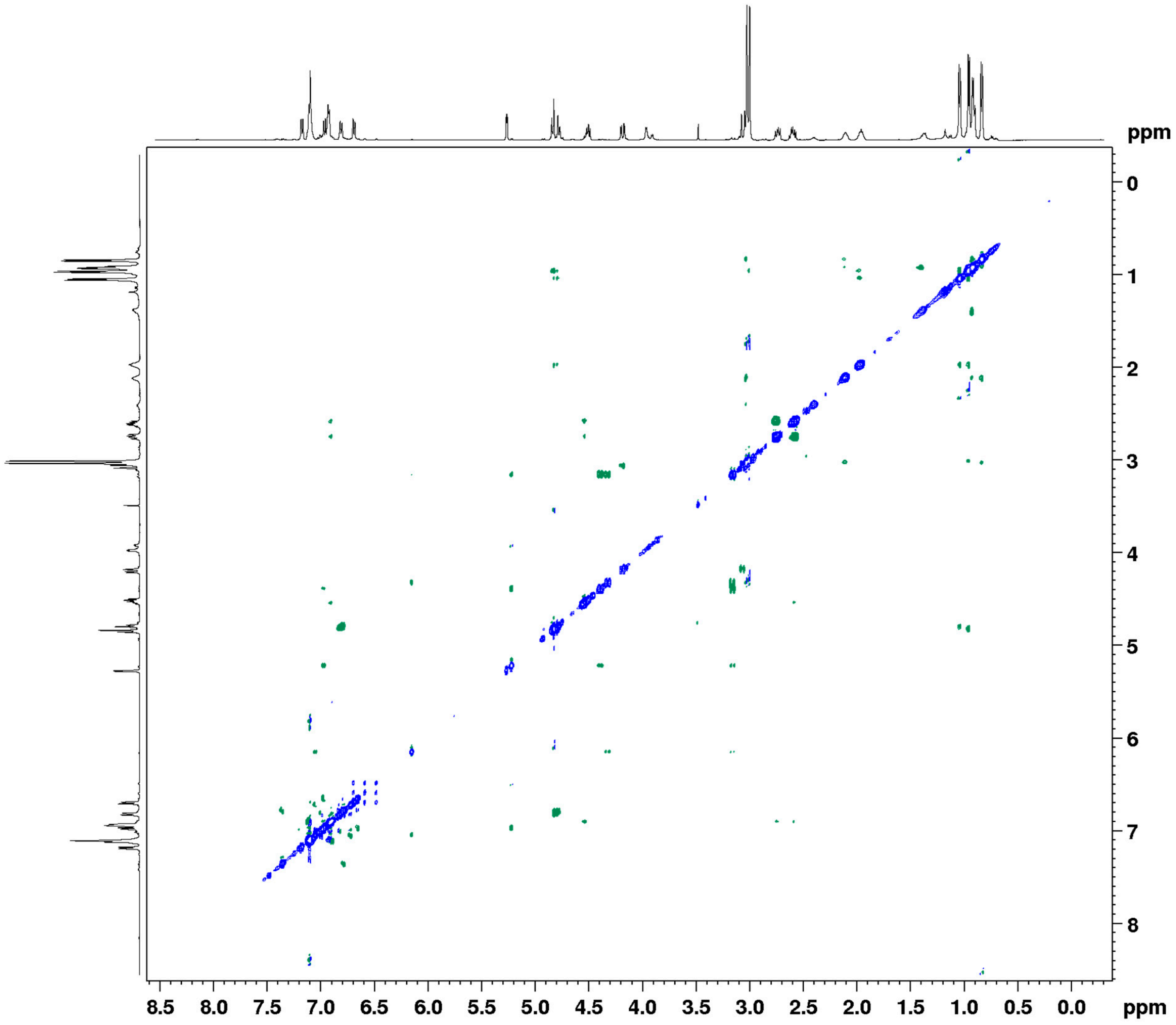

Figure S14. ROESY (TFA-*d*, 500) spectrum of pandamine (**1**).

(A)

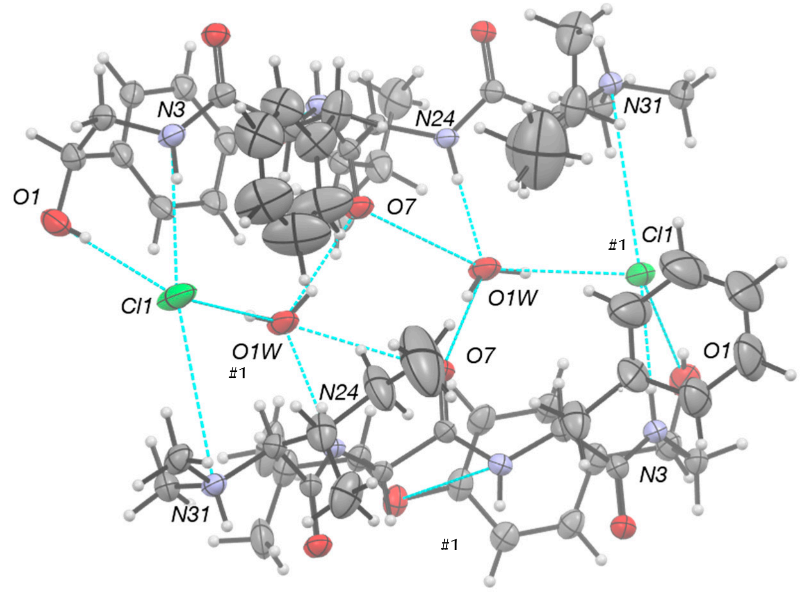

(B)

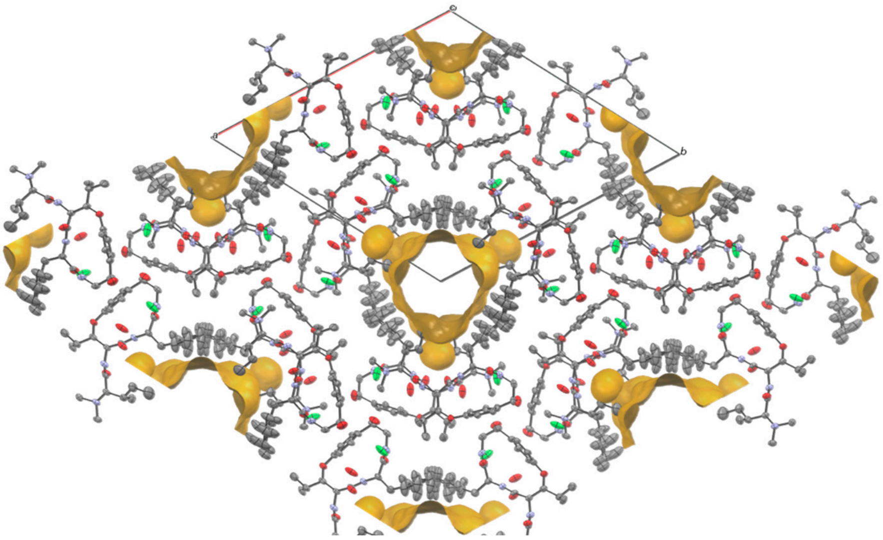

(C)

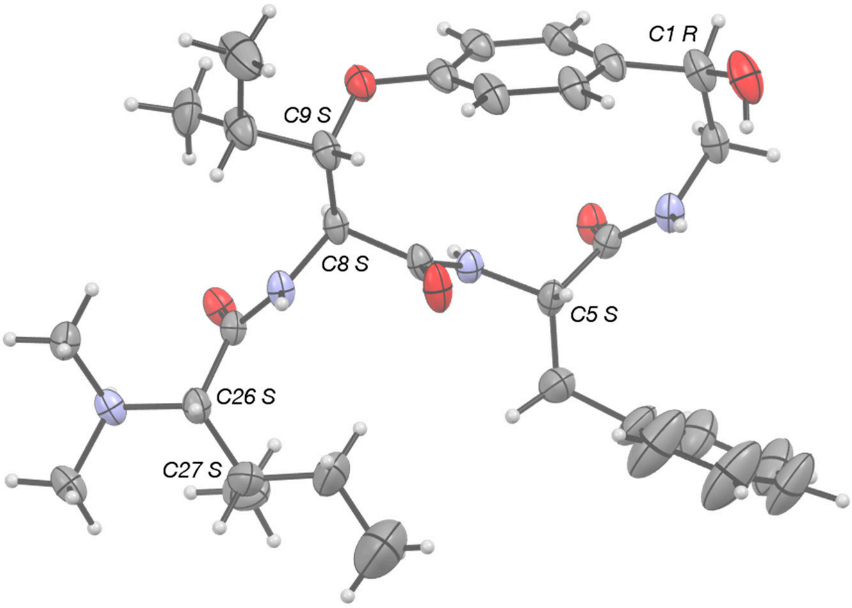

(D)

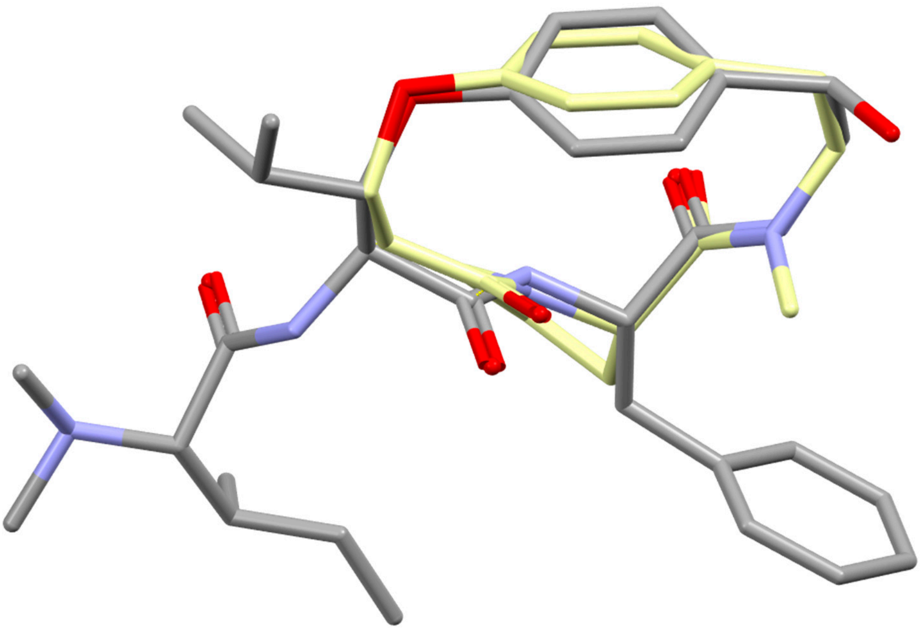

Figure S15 (**A**) Partial view of the unit cell of **1** showing the head-to-tail dimer indirectly H-bonded *via* two waters and two chlorine anions (symmetry transformation used to generate the pandamine molecule in the bottom and the water and chlorine marked by #1 y, x, 1-z). (**B**) Partial view of the crystal **1** packing down the ***c*** axis. The solvent-accessible voids are delineated by yellow surfaces. (**C**) Ortep view of the pandamine cationic form with the labelling of chiral centers (Displacement ellipsoids are drawn at the 50% probability level). Only the major conformer of the phenyl group is shown for clarity. (**D**) Overlay of Pandamine over the macrocycle of CSD RefCode BUCKOZ (carbon atoms in yellow) (the RMSD of the 12 common atoms is 0.252Å).

Table S1 Crystal data and structure refinement for pandamine (**1**).

Identification code	pandamine (**1**)	
Empirical formula	$[C_{31}H_{45}N_4O_5]^+$, Cl^-, H_2O [+solvent]	
Formula weight	607.1	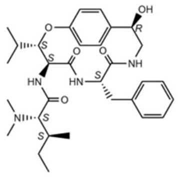
Temperature	123.0 (2) K	
Wavelength	0.71073 Å	
Crystal system	Trigonal	
Space group	$P3_221$	
Unit cell dimensions	a = 23.0122(13) Å	α= 90°.
	b = 23.0122(13) Å	β= 90°.
	c = 11.8273(6) Å	γ = 120°.
Volume	5424.2(7) $Å^3$	
Z	6	
Density (calculated)	1.115 Mg/m^3	
Absorption coefficient	0.148 mm^{-1}	
F(000)	1956	
Crystal size	0.22 x 0.05 x 0.05 mm^3	
θ range for data collection	2.470 to 27.101°.	
Index ranges	$-29 \leq h \leq 28$, $-29 \leq k \leq 29$, $-15 \leq l \leq 14$	
Reflections collected	47767	
Independent reflections	7989 [R(int) = 0.0501]	
Completeness to θ = 25.242°	99.7 %	
Absorption correction	Semi-empirical from equivalents & Gaussian	
Max. and min. transmission	1.000 and 0.854	
Refinement method	Full-matrix least-squares on F^2	
Data / restraints / parameters	7983/ 507 / 505	
Goodness-of-fit on F^2	1.047	
Final R indices [$I > 2\sigma(I)$]	R1 = 0.0445, wR2 = 0.1149	
R indices (all data)	R1 = 0.0525, wR2 = 0.1195	
Absolute structure parameter	-0.02.(2) §	
Largest diff. peak and hole	0.268 and -0.238 e.$Å^{-3}$	
CCDC deposit number	2235441	

§Flack x determined using 2859 quotients [(I+)-(I-)]/[(I+)+(I-)].
